# How 'idiopathic' is adolescent idiopathic scoliosis? A systematic review on associated abnormalities

**DOI:** 10.1186/1748-7161-10-S1-P5

**Published:** 2015-01-19

**Authors:** Tom P Schlösser, Geert J van der Heijden, Anne L Versteeg, René M Castelein

**Affiliations:** 1 Department of Orthopaedic Surgery, University Medical Center Utrecht, Utrecht, the Netherlands; 2 Department of Epidemiology, University Medical Center Utrecht, Utrecht, the Netherlands; 3 Department Social Dentistry, Academic Center for Dentistry Amsterdam, VU Amsterdam University and Univers, the Netherlands

## Objectives

Despite more than a century of dedicated research, the etiology and pathogenesis of AIS remain unclear. By definition, 'idiopathic' implies an unknown cause. Nevertheless, many abnormalities concomitant to AIS have been described, often with the suggestion that these abnormalities are related to etio-pathogenesis. Insight in the concomitant abnormalities may assist in improving the understanding of the etiological pathways of AIS. For understanding of the etiology of adolescent idiopathic scoliosis (AIS), this systematic review gives as complete an overview as possible of abnormalities that have been implicated to be concomitant with AIS.

## Methods

Original studies comparing untreated AIS patients with healthy adolescents on abnormalities other than the deformity of the spine were retrieved from PubMed and Embase. We followed PRISMA guidelines and to quantify the relationship between each abnormality and AIS we used a best-evidence-syntheses for relating risk-of-bias to consistency of effect sizes.

## Results

We identified 88 relevant citations, forty-seven carried high risk-of-bias and twenty studies did not report quantitative data in a sufficient manner. The remaining twenty-one publications failed to report data from before initiation of the deformity and blind assessments. These cross-sectional studies provided data on fourteen abnormalities concomitant to AIS. With our best-evidence-syntheses we were unable to find both strong evidence and a consistent pattern of occurrence for AIS and any of these abnormalities. From moderate risk-of-bias studies a relatively consistent pattern of occurrence for AIS and impaired gait control (4 studies; 155 subjects; Cohen's d= 1.00) and decreased bone mineral density (2 studies; 954 subjects; Cohen's d= -0.83) was found. For nine abnormalities a consistent pattern of occurrence with AIS was found, but the evidence for these was weak.

## Conclusions

Based on the available literature, strong evidence is lacking for a consistent pattern of occurrence of AIS and any abnormality. In addition, abnormalities were never studied before the onset of the deformity. The relevance for understanding the multifactorial etiology of AIS is very limited.

